# Supplementary figures and images for: Identification and Functional Analysis of AopN, an Acidovorax Citrulli Effector that Induces Programmed Cell Death in Plants
Source: Int J Mol Sci. 2020 Aug 22;21(17):6050. doi: 10.3390/ijms21176050 (PMC7504669; doi:10.3390/ijms21176050)

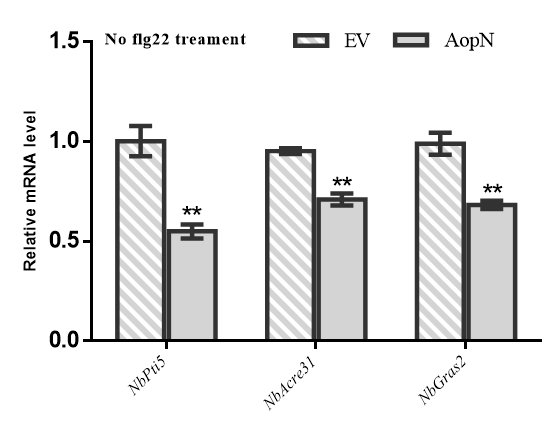

Supplement: Supplementary file 1 [file ijms-21-06050-s001.zip › ijms-877174-proofed-supplementary/Figure S1.png]

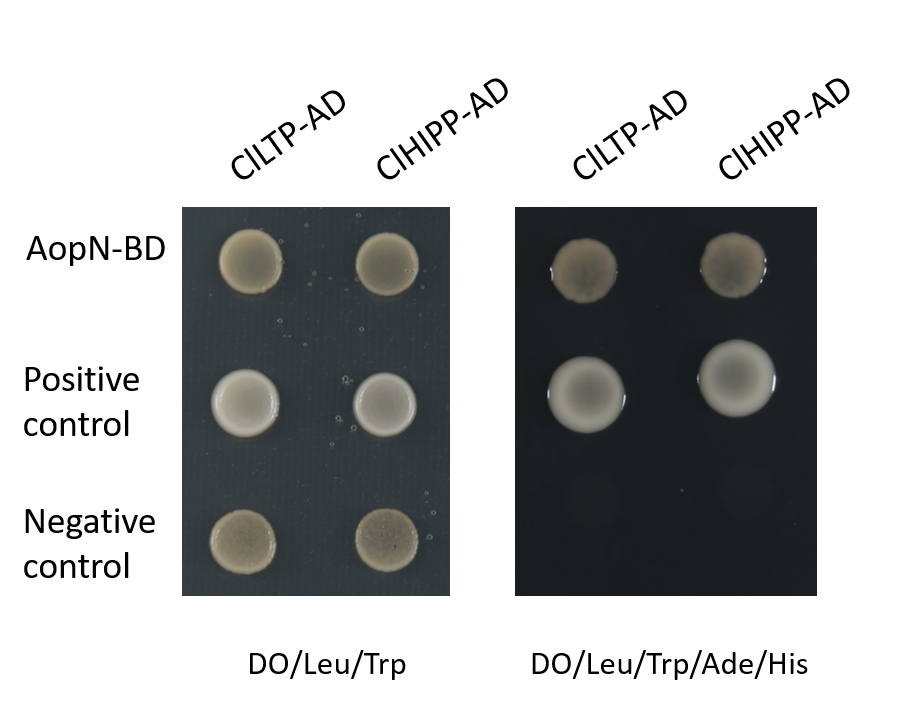

Supplement: Supplementary file 1 [file ijms-21-06050-s001.zip › ijms-877174-proofed-supplementary/Figure S2.png]
